# Supplementary material for: Investigation of an outbreak of acute algal-associated dermatoses among artisanal fishermen in Senegal: A one health approach
Source: PLOS Glob Public Health. 2026 Apr 24;6(4):e0006328. doi: 10.1371/journal.pgph.0006328 (PMC13108758; doi:10.1371/journal.pgph.0006328)
Supplement: S1 Appendix — (DOCX) [file pgph.0006328.s001.docx]

**S1 Appendix**: Laboratory Protocols and Validation

1. Sample preparation

- Environmental samples (seawater): Water samples were filtered on 0.8 μm polycarbonate membranes to concentrate phytoplankton cells. The samples were rinsed before extraction.

2. Analytical instruments and methods

- Mass spectrometry: Use of a liquid chromatography system coupled with tandem mass spectrometry (LC-MS/MS) for the detection of toxins (Portimine A).

3. Detection limits and quality control

- Detection Limits (LOD): The detection limits for Portimine A by LC-MS/MS have been established according to international seafood safety standards.
- Quality controls: Each set of analyses included procedure blanks and spiked samples to verify recovery rates.
