# Supplementary material for: Investigation of an outbreak of acute algal-associated dermatoses among artisanal fishermen in Senegal: A one health approach
Source: PLOS Glob Public Health. 2026 Apr 24;6(4):e0006328. doi: 10.1371/journal.pgph.0006328 (PMC13108758; doi:10.1371/journal.pgph.0006328)
Supplement: S2 Appendix — (DOCX) [file pgph.0006328.s002.docx]

**S2 Appendix**: Toxin Detection, Organism Identification and Chain of Custody

1. Organism Identification Techniques

- PCR (Polymerase Chain Reaction): Use of genomic amplification to target specific regions of rDNA (LSU, ITS) to confirm the identity of the *Vulcanodinium rugosum species*.
- Reference Standards: The resulting sequences were compared to reference strains deposited in international databases (GenBank).

2. Methods for Detecting Toxins

- Extraction: Methanol extraction of biological and environmental samples.
- Toxin analysis: Specification of the detection of Portimine A, a characteristic metabolite of *V. rugosum*, by LC-MS/MS in MRM (Multiple Reaction Monitoring) mode.
- Validation: Procedures validated by comparing retention times and fragment ion ratios with Portimine A standards.

3. Chain of custody and storage

- Chain of custody: A strict traceability record was maintained from sampling at fishing sites to receipt to national laboratories and final transfer.
- Storage conditions: * Samples kept in the dark and kept at +4°C during local transport, then frozen at -20°C for international shipping.
