# Supplementary material for: Investigation of an outbreak of acute algal-associated dermatoses among artisanal fishermen in Senegal: A one health approach
Source: PLOS Glob Public Health. 2026 Apr 24;6(4):e0006328. doi: 10.1371/journal.pgph.0006328 (PMC13108758; doi:10.1371/journal.pgph.0006328)
Supplement: S3 Appendix — (DOCX) [file pgph.0006328.s003.docx]

**S3 Appendix**: Psychosocial and Professional Impact

| Key Themes | Illustrative Quotes |
| --- | --- |
| Stigmatization and COVID-19 | "People avoided us at the dock... they thought we were bringing a new form of COVID-19." |
| Financial Impact | "Without fishing, there is no money to feed the family or pay school fees." |
| Uncertainty and Fear | "We were afraid it might be contagious for our wives and children at home." |
| Therapeutic Measures | "Some used salt or plants, which sometimes worsened the burns." |
