# Supplementary material for: Investigation of an outbreak of acute algal-associated dermatoses among artisanal fishermen in Senegal: A one health approach
Source: PLOS Glob Public Health. 2026 Apr 24;6(4):e0006328. doi: 10.1371/journal.pgph.0006328 (PMC13108758; doi:10.1371/journal.pgph.0006328)
Supplement: Attachment — Submitted filename: Comments on Dermatoses Paper.docx [file pgph.0006328.s004.docx]

Congratulations on carrying out this outbreak investigation as well as sharing it as a manuscript!

Here some thoughts that I have on the manuscript:

What is the baseline rate of dermatosis in this community? Did you find any information or not? Please report in either situation.

It would be good to illustrate in a diagram or a narrative, how many patients were recruited for each of the stages of the data collection.

Ex:

- Number of patients identified through document review
- Number of patients who participated in bacteriological and virological examination
- Number of patients who participated in anatomopathological examinations
- Number of patients participated in environmental survey

Also, it may be good to clearly illustrate the data collection tools used and methods deployed in each stage.

| No | Component | Tools used |
| --- | --- | --- |
| 1 | Document review | Ex: Data collection format  Line list |
| 2 | bacteriological and virological examination | Provide details here |
| 3 | Anatomopathological examination | Provide details here |
| 4 | Environmental survey and sampling | Provide details here |

Since this is an outbreak investigation report, it is good to use some format to cover the standard steps of an outbreak investigation. For example, steps provided here can be used as a guideline. Make sure it or any other source is referred and used in writing the methodology.

<https://archive.cdc.gov/www_cdc_gov/csels/dsepd/ss1978/lesson6/section2.html#:~:text=Arrange%20to%20bring%20a%20laptop,is%20likely%20to%20be%20lengthy>.

It is essential to state your hypothesis about the etiological agent, as well as how you reject, fail to reject it with evidence.

Line 1: Algal origin: Isn’t it better to remove this from the title as the reader would be motivated to read the paper to find out the possible etiological agent, “the mystery as you rightly stated in Line 299, in the abstract or the paper than presenting it in the title. Just a suggestion.

Line 36, line 188 – case fatality rate of 0%: Better to state as no deaths were reported.

Figure 2: Period is given as March 2025. What does this mean? Shouldn’t the period be the study period?

Line 237 – 240 – I think these details can go into the discussion. Under the methods, please specify where the toxicological tests were performed.

Lines 256 – 262: How did you find this information? Did you have any open-ended questions? I do not see any numbers or proportions here unlike in other sections.

Lines 263 – 286: How did you come up with these results? I do not see notion of the methods used to arrive at these results under the methods section. Please make sure that these findings align with the methods used. In another words, mention in the methods section, how you came up with the results section on strategies and measures taken to control the outbreak.

Lessons learned Lines 287 – 298 Best place as I see would be the discussion.

Line 299

It is essential that you logically and clearly prove in a step-by-step manner how you came into your etiological conclusion. Explain how any other etiological agents could be excluded.

Lines 318 – 319 – In the results section elaborate this before discussing here with the use of Figure 2.

388-390 It may be good to use language such as “likely cause”.
