# Supplementary material for: Investigation of an outbreak of acute algal-associated dermatoses among artisanal fishermen in Senegal: A one health approach
Source: PLOS Glob Public Health. 2026 Apr 24;6(4):e0006328. doi: 10.1371/journal.pgph.0006328 (PMC13108758; doi:10.1371/journal.pgph.0006328)
Supplement: Attachment — Submitted filename: Response to Reviewers.docx [file pgph.0006328.s006.docx]

**Dr. Mbouna Ndiaye**

**MD, MPH, Field Epidemiologist**

Field Epidemiology Training Program (FETP) Coordinator,

Dakar, Senegal

Phone (+221) 651 94 85

Watsap: (+221) 70 960 74 23

[mbounandiaye@hotmail.fr](mailto:mbounandiaye@hotmail.fr)	Dakar, 12 Sept 2025

**Response Letter to Reviewers**

**Objet** : Response to reviewers

*Ref.: PGPH-D-25-01888*

*«* **investigation of an outbreak of unusual acute dermatoses of algal origin among fishermen in Senegal** *»*

We provide you with point-by-point responses to the concerns raised in the form of a table (see below).

We have attached to this letter:

- An annotated copy of the manuscript that highlights the changes made to the original version. It was uploaded in a separate file entitled " **Revised Manuscript with Track Changes** ".
- An unmarked version of my revised document with no track of changes. It was uploaded in a separate file entitled "**Manuscript**".
- A complete data availability statement
- Two source files of the data.

| **Issues raised** | **Actions taken**  **Nota Bene: the lines mentioned in this column refer to the new corrected version entitled "Manuscript"** |
| --- | --- |
| **Review requirements:** | |
| Please provide a complete statement of data availability in the submission form, making sure to include any necessary access information or a reason why you cannot make your data freely accessible. | A complete statement of data availability has been attached to the submission form |
| Potential copyright issues:a) Figure 2 | Figure 2 (map) has been reproduced; The base layer has been replaced and does not pose any copyright issues |
| Figures 3 and 4 include an image of an identifiable person. Please provide written confirmation or a release form, signed by the subject(s) (or their parents/legal guardians), authorizing the photography and publication of their images under our CC-BY 4.0 license. | The identifiable person image has been removed (Figure 3) and obscured (Figure 4) |
| **Reviewer's comments:** | |
| 3. Have the authors made all the data supporting the results of their manuscript fully accessible? PLOS [Data's policy](https://journals.plos.org/globalpublichealth/s/data-availability) requires authors to make fully accessible, without restriction, all data supporting the results described in their manuscript, with rare exceptions. The data must be provided with the manuscript or its additional information, or deposited in a public repository. For example, in addition to summary statistics, data underlying means, medians, and variance measures must be available. Any restrictions on public sharing of data (e.g. participant privacy or use of third-party data) should be specified. | All data are available in the manuscript. I am sending you 2 source files of the data. |
| L56 to 71, Minor Viewpoint: This section focuses excessively on general topics instead of quickly addressing the topic of specific interest, namely the seaweed dermatoses discussed in the article. While it may be helpful to start with background information before getting into the details, it's important to keep it concise by only briefly mentioning these general aspects in the introduction. Unfortunately, this section does not achieve that balance. | Correction made to achieve the desired balance (line 55 -86) |
| The introduction was written after the analysis, which detracts from the suspense until the results are revealed (L234). The general aspects I mentioned earlier, such as pollution, chemical agents, allergens and bacterial agents, should be presented in the form of hypotheses | Indeed, the introduction was updated after the investigation.  Correction made: assumptions have been made (lines 135 to 138) |
| L77 to 83: Shouldn't this section be grouped into a single paragraph with lines 74 to 76? | Correction made: line 71 |
| L31, L129: Question: In what way is this study multicenter? It would be useful to provide additional details to explain the use of this term. | I remove the word, as it is confusing (line 122). I wanted to say that the investigation was carried out in all the health districts in the region. |
| L142 to 152: It would be useful to clarify whether all participants were eligible for the different parts of the research or whether there was a specific eligibility algorithm for each section.  Important details are missing in the methodology section. There is a gap in sampling. Key aspects to be clarified include the estimated sample size and the participant selection process. How many topics were included? Have all the health facilities in the study region been selected, or only some? If only some, what criteria were used? Were health facilities the only way to identify participants, or were there other methods? Once identified in healthcare facilities, how were these participants approached, especially for non-hospitalized patients? Were all eligible cases included, or were some identified but excluded from the study for specific reasons? | The case definition allowed for patient recruitment. Not all individuals who met the case definition were considered in the survey. We do not know how many  Correction made: clarification has been made on the choice of participants in each part of the research: Line 139 to 148  There is no calculation of the size of a sample. All patients seen in health facilities and meeting the case definition are only considered. All health facilities that received patients are concerned by the study. |
| L188-196: It is important to specify whether the phenomenon is new, i.e. it did not exist before in the region and has just emerged, or whether it existed before but on a smaller scale. Neither this paragraph nor those between lines 87-93 or 299-300 provide specific information. | The clarification has been made as you suggested (lines 296 and 297) |
| L210: "All the patients in the study were men who worked as fishermen." The lack of detail on the sampling technique raises concerns about possible selection or information bias. The definition of the population studied (lines 131-137) also raises questions, particularly given the fact that this occupation is predominantly male, as indicated by the criteria stating: "... who had been at sea in the five days prior to the onset of symptoms,... ». In addition, skin lesions in the case definition may exclude other skin diseases affecting both sexes. In an epidemiological study, the inclusion criteria must be sensitive, while the diagnostic criteria must be specific. It is important to distinguish between the characteristics of the subjects being evaluated (ideally including both males and females) and those of the confirmed cases (which may be only males). I recommend that the authors provide a flow chart detailing the individuals assessed, including the 555 individuals reported ill, while specifying the gender distribution and grounds for exclusion at each stage. | We have circumscribed our field of study because from the outset we know that the common problem of all is the sea.  So our investigation was only interested in people with dermatological lesions who had been at sea in the five days prior to the onset of symptoms or who had been in contact with marine equipment. The selection was not made according to sex. But it turned out that it was only men who met the case definition. We do not have information on the number of people not included. We accept that this is a selection bias, but the resources did not allow us to expand the study population.  We will point this out as a limitation in our study. |
| L224. These photos probably show lesions that are still active during the investigation. Given the 0% mortality rate, what were the possible outcomes? If recovery was a possible outcome, what was the average duration of the disease? Were there any complications and how were they treated? If data are available, it would be useful to compile these results into a single section (e.g., L283-286) for a clearer overview of clinical aspects (L211). In addition, the authors should clarify in the methodology how the differential diagnosis of dermatoses was made and who was responsible for it. | No deaths have been reported to our knowledge. We do not have exact information on the average duration of the disease.  Severe cases were hospitalized. For complicated cases of bacterial superinfection, antibiotic treatment was administered; This was specified on line 282. A multidisciplinary team including dermatologists, infectious disease specialists... was responsible for the management of the differential diagnosis (see lines 262 and 263). |
| L228 I have part of the answer to my question: "Were all participants eligible for all parts of the study?" It seems that this is not the case. Therefore, my observation remains valid: this aspect should be clearly specified in the methodology. In addition, the criteria for selecting these 10 patients or determining eligibility for a part of the study should also be described. Regarding lines 255 to 262 and 263 to 286: Are there any figures or tables to support these results? Is this section based on a qualitative approach? If so, the methodology should specify that both quantitative and qualitative approaches were used. | I corrected this by specifying how the samples were chosen in the "collection technique" section.  There are no tables or figures for these results.  Yes, this section is based on a qualitative approach. I corrected by adding precision in the methodology (lines 149 to 152) |
| What is the baseline rate of dermatoses in this community? Did you find any information or not? Please report it in both cases. | Occupational dermatoses are very little studied. We do not have statistics in this community (Lines 76 and 77) |
| It would be useful to illustrate, using a diagram or narrative, how many patients were recruited at each stage of data collection. | I corrected this by specifying how the samples were chosen in the "collection technique" section.  This has been illustrated in the form of narration (Line 139 – 148) |
| It is essential to state your hypothesis regarding the etiologic agent, as well as explain how you reject or do not reject it, with supporting evidence. | Correction made: assumptions made: line 135- 138  We looked for several possible causes that justify the bacteriological, virological, histopathological and toxicological examinations carried out |
| Since this is an outbreak investigation report, it is recommended that you use a format that covers the standard steps of an investigation of this type. For example, the steps suggested here can serve as a guide. | We used the standard steps of an investigation in the process, but they remain implied in the report:  For example:  Step 1: prepare: meeting with the authorities (lines 163 and 164)  Step 2: Confirm the existence of the epidemic: line 81-92  Step 3: Define the cases: line 127  Step 4: Describe the cases in time and person: result chapter (From line 185)  Step 4, 5, 6, etc.... |
| Line 36, line 188 – case fatality rate of 0%: It would be better to indicate "no deaths were reported". | Correction made on line 188 |
| Figure 2: The time period shown is March 2025. What does that mean? Shouldn't it be the study period? | This is the period of creation of the map.  I removed it if it causes any confusion |
| Line 237 – 240: I think these details can go into the discussion.  In the methods section, specify where the toxicological tests were carried out. | Correction made. Details have been moved to the discussion section (see Line 342 – 344)  The toxicological tests were carried out in France: clarification made in the methodology chapter: line 148 |
| Lines 263 – 286: How did you arrive at these results? I don't see the mention of the methods used to get these results in the methods section. Please ensure that these results are consistent with the methods used. In other words, indicate in the methods section how you obtained the results regarding the strategies and measures to control the epidemic. | Correction made  I corrected by specifying in the methodology section that a qualitative survey was also carried out to obtain the results concerning the strategies and measures for controlling the epidemic Line 149 to 152 |
| Lessons learned lines 287 – 298: In my opinion, the best place would be in the discussion. | In fact, I left them in the results part because they are also results of the qualitative survey. As a team, we discussed the position of the lessons learned. Some members suggested that they should be included in the results chapter instead of being included in the discussion. But, if you insist, I can move them. |
| Line 299  It is essential to demonstrate logically and clearly, step by step, how you arrived at your etiological conclusion. Explain how other etiologic agents could be ruled out. | We looked for several possible causes that justify the bacteriological, virological, anatomopathological and toxicological examinations performed.  It was the bio-toxicological results of the examinations that made it possible to determine the probable cause |
| Lines 318 – 319 – In the results section, expand on this point before discussing it here with Figure 2. | The epidemic mainly affects populations originating from the Senegalese coast in coastal areas known for their fishing activities (Fig 2). This is the only relevant result from the map. This clarification was made on line 203-204 |
| Lines 388-390: It would be better to use language such as "probable cause." | Correction made (line 389) |
