# Supplementary material for: Investigation of an outbreak of acute algal-associated dermatoses among artisanal fishermen in Senegal: A one health approach
Source: PLOS Glob Public Health. 2026 Apr 24;6(4):e0006328. doi: 10.1371/journal.pgph.0006328 (PMC13108758; doi:10.1371/journal.pgph.0006328)
Supplement: Attachment — Submitted filename: Response_to_Reviewers_auresp_2.docx [file pgph.0006328.s008.docx]

**Dr. Mbouna Ndiaye**

**MD, MPH, Field Epidemiologist**

Field Epidemiology Training Program (FETP) Coordinator,

Dakar, Senegal

Phone (+221) 651 94 85

Watsap: (+221) 70 960 74 23

[mbounandiaye@hotmail.fr](mailto:mbounandiaye@hotmail.fr)	Dakar, 31 Oct 2025

**Response Letter to Reviewers**

**Objet** : Response to reviewers

*Ref.: PGPH-D-25-01888*

*«* **investigation of an outbreak of unusual acute dermatoses of algal origin among fishermen in Senegal** *»*

We provide you with point-by-point responses to the concerns raised in the form of a table (see below).

We have attached to this letter:

- An annotated copy of the manuscript that highlights the changes made to the original version. It was uploaded in a separate file entitled " **Revised Manuscript with Track Changes** ".
- An uncorrected version of my revised document with no track of changes. It was uploaded in a separate file entitled "**Manuscript**".

| **Issues raised** | **Actions taken**  **Nota Bene: the lines mentioned in this column refer to the new corrected version entitled "Manuscript"** |
| --- | --- |
| Consider changing the title to: «Investigation of an outbreak of acute algal-associated dermatoses among artisanal fishermen in Senegal: a one health approach» | Modification accepted on the title |
| Add a sentence summarizing the public health implications in the conclusion. | I do not quite understand that suggestion. By adding a sentence, I am afraid of repeating myself. I think that several public health implications were put forward in the conclusion in the form of public health actions or measures to be taken. |
| Case Identification and Sampling: Please include a flow chart showing how the 555 cases were identified, screened, and included/excluded in different components (epidemiological, laboratory, qualitative).  Clarify whether cases were identified solely by health facilities or also through community outreach.  Discuss how the case definition might have excluded atypical or mild cases and introduce this as a limitation. | Organizational chart has been added (Figure 5)  The cases were identified only in health facilities. This clarification was made in the collection procedure (line 170-171) and in the results (line 191-192)  A limitation chapter has been added at the end of the Discussion chapter (line 394 -399) |
| Population Denominator and Attack Rate: Justify the use of the regional population as the denominator of the attack rate. Provide an alternative calculation using the population of anglers exposed (e.g., 10,200 anglers) and report both rates.  Explicitly remove the numerator/denominator values in the Results section and report only percentages. | Initially, we started with the assumption that the entire population is at risk. Given that only fishermen are sick, I agree to reconsider the denominators by taking into account only fishermen. Therefore, I have taken the calculations by taking into account only the fishermen who are at risk (line 197)  Correction made |
| Selection Criteria for Laboratory Testing: Clearly describe how the 10 bacteriovirology samples, 4 biopsies, and 8 environmental samples were selected. Indicate whether these were chosen randomly, based on convenience or severity.  Provide details of the laboratory's protocols (sample preparation, instruments, analytical methods, detection limits, quality controls, inter-laboratory validation with IFREMER). | It was indicated in the text that the choice was made at random.  A selection flowchart has been added to the Manuscript (Figure 5)  I don't have this information, and I'm not a biologist by training.  Another manuscript on aspects of biotoxicology is being processed by specialists in the field.  I must point out that IFREMER is a French public research institute entirely dedicated to understanding the ocean. Ifremer is involved in scientific initiatives and programmes of national, European and international scope.  More information: https://www.ifremer.fr/fr |
| Toxicology and Identification of Vulcanodinium rugosum: Develop methods for the detection of toxins and identification of organisms: specify techniques (e.g., LC-MS/MS, microscopy, PCR), reference standards, and validation procedures. Clarify the chain of custody and storage conditions for samples. | Additional details have been provided (line 148-151) |
| Interpret results with caution – rephrase causal claims as "probable algal origin" or "evidence consistent with" rather than definitive causation. | This clarification was made in the conclusion of the study (line 402) |
| Statistical and epidemiological reports: Ensure that all tables and text include 95% confidence intervals and p-values for effect estimates (for example, use of PPE in relation to severity). Check for consistency between numbers in the abstract, results, and tables. If applicable, indicate the duration of illness, hospitalization rate, and clinical outcomes; otherwise, indicate that the data has not been saved. | Audits have been completed |
| Qualitative component: Include a brief subsection on qualitative methods: describe the selection of participants, the interview/focus group process, data transcription, coding, and the method of thematic analysis. | Additional details have been provided (line 155-157) |
| Present a short table summarizing key themes or illustrative quotes related to psychosocial and professional impact. | This has been presented in the section on the impact of dermatoses (line 259-266) |
| Discussion and interpretation: Avoid repetition of results. Further the analysis by explaining why the outbreak occurred, drawing parallels with similar events (e.g., Cuba). Discuss the possible biological mechanisms of portimine A and environmental triggers. | Effective consideration |
| Recognize and develop the limitations of the study (sampling bias, lack of quantification of exposure, recall bias, lack of control group). | A limitation chapter has been added at the end of the Discussion chapter (line 394 -399). |
| Ethical Considerations and Image Permissions: Provide full details of the ethics approval (name, number, and date of the IRB). Indicate how informed consent was obtained (and parental consent if minors are included). Confirm that patient images have been masked/anonymized and used with consent or removed appropriately. | The study does not raise ethical problems as was pointed out in the Ethical Considerations (lines 184 to 189). The ethical approval was attached in the electronic submission system of the PLOS journal: N°000698/MSAS/DGS/DP of October 09, 2020.  The images were masked/anonymized without any possibility of identifying the people |
| Data Availability: -Provide a complete statement of data availability as required by PLOS: Indicate where the dataset and supporting files are accessible (repository name, DOI/URL). If access is restricted, provide the reason and contact person for the data requests. | Statements made and supporting files attached in the PLOS electronic submission system |
| Tables, figures, and formatting: Make sure each table has clear titles; defined abbreviations; Correct totals and percentages; Appropriate footnotes explaining subsamples (e.g., biopsy, bacteriology); Include figure captions indicating data sources, map copyrights, and image consent. Standardize formatting across the board (fonts, heading levels, spacing). | Effective consideration |
| Language and style: Simplify long sentences, especially in the introduction and discussion. Ensure consistent use of tenses (Methods in the past tense; Results in the present; Discussion in the past/present). | Effective consideration |
| References: Update the literature to include recent work (2020-2024) on harmful algal blooms, exposure to marine toxins, and skin toxicity. Standardize all references according to the PLOS style (authors, year, title, journal, DOI). Verify that all citations in the text match the reference list. | Effective consideration |
