# Supplementary material for: Investigation of an outbreak of acute algal-associated dermatoses among artisanal fishermen in Senegal: A one health approach
Source: PLOS Glob Public Health. 2026 Apr 24;6(4):e0006328. doi: 10.1371/journal.pgph.0006328 (PMC13108758; doi:10.1371/journal.pgph.0006328)
Supplement: Attachment — Submitted filename: Dermatoses comments.docx [file pgph.0006328.s007.docx]

• Consider revising the title to: “Investigation of an outbreak of acute algal-associated dermatoses among artisanal fishermen in Senegal: A One Health field investigation.” – Addressed.

• Add a sentence summarizing public health implications in the Conclusion.

This has not been addressed. Suggest adding something similar to this note:

“From a public health perspectives, this study highlights how effective surveillance coupled with evidence informed action, coupled with community awareness can contribute to the reduction of impacts of outbreaks at the intersection of environmental and occupational health.”

• Case Ascertainment and Sampling: Please include a flow diagram showing how the 555 cases were identified, screened, and included/excluded in different components (epidemiologic, laboratory, qualitative).

This has been addressed.

Clarify whether cases were identified only through health facilities or also through community-based outreach. Discuss how the case definition might have excluded atypical or mild cases and introduce this as a limitation.

This has been addressed.

• Population Denominator and Attack Rate: Justify the use of the regional population as the denominator for the attack rate. Provide an alternative calculation using the exposed fishing population (e.g., 10,200 fishermen) and report both rates.

This comment has not been addressed. Please add something like this:

| Attack rate with regional population as the denominator | Attack rate with exposed fishing population as the denominator |
| --- | --- |
| Please do the calculation and report. | 5.4% |

Delete the numerator/denominator values explicitly in the Results section, and report only the percentages.

This comment has not been addressed.

Ex: A total of 555 cases were diagnosed with an attack rate of 5.4% (555/10,200) among fishermen and no deaths were reported.

This should be corrected as

A total of 555 cases were diagnosed with an attack rate of 5.4% among fishermen and no deaths were reported.

• Selection Criteria for Laboratory Testing: Clearly describe how the 10 bacterio-virology samples, 4 biopsies, and 8 environmental samples were selected. Indicate whether these were randomly chosen, convenience-based, or severity-driven.

This comment has been addressed.

Provide details of the laboratory protocols (sample preparation, instruments, analytical methods, detection limits, quality controls, inter-laboratory validation with IFREMER).

This has not been addressed. Suggest adding an annexure with these details.

• Toxicology and Identification of Vulcanodinium rugosum: Expand on the methods for toxin detection and organism identification: specify techniques (e.g., LC-MS/MS, microscopy, PCR), reference standards, and validation procedures. Clarify the chain of custody and storage conditions for samples.

See my comment above. These details can be added as an annexure. This will increase the rigor of your paper and usefulness to those who may want to follow the same steps during another outbreak.

• Interpret results cautiously—rephrase causal claims to “probable algal origin” or “evidence consistent with” rather than definitive causation.

This has been addressed with appropriate langue “probable cause”.

• Statistical and Epidemiological Reporting: Ensure all tables and text include 95% confidence intervals and p-values for effect estimates (e.g., PPE use vs. severity).

This has been partially addressed. Suggest adding confidence interval to Table 1, for example.

Verify consistency between numbers in Abstract, Results, and Tables. If available, report disease duration, hospitalization rate, and clinical outcomes; otherwise, state that data were not recorded.

This has been addressed.

• Qualitative Component: Include a brief Qualitative Methods subsection: describe participant selection, interview/focus group process, data transcription, coding, and thematic analysis method.

Following aspects have not been addressed: data transcription, coding, and thematic analysis method.

Please mention at least briefly.

• Present a short table summarizing key themes or illustrative quotes related to psychosocial and occupational impact.

This comment has not been addressed.

• Discussion and Interpretation: Avoid repetition of results. Deepen analysis by explaining why the outbreak occurred, drawing parallels with similar events (e.g., Cuba). Discuss possible biological mechanisms of portimine A and environmental triggers.

This has been addressed.

• Acknowledge and elaborate on study limitations (sampling bias, lack of exposure quantification, recall bias, absence of control group).

Some of these have been addressed.

Please address the following as suggested by the reviewer:

Lack of exposure quantification, recall bias

• Ethical Considerations and Image Permissions: Provide full details of ethical approval (IRB name, number, and date). State how informed consent was obtained (and parental consent if minors are included). Confirm that patient images were obscured/anonymized and used with consent or appropriately removed.

This has been partially addressed.

I assume IRB approval was not feasible due to the emergency nature of the study. In that case, please state that IRB approval was not feasible. If it was obtained, please state from where and the number. The study being commissioned by the Ministry of Health does not remove the need for IRB approval. However, if this was part of routine service provision, probably you could justify this to some extent. Please have a sentence specifically about the photographs. “Patient images were obscured when the identification details were visible. Consent was obtained for presenting photographs in unidentifiable format for educational purposes. If this was not done, explain why it was not done.

• Data Availability:-Provide a complete Data Availability Statement as required by PLOS: State where the dataset and supporting files can be accessed (repository name, DOI/URL). If access is restricted, specify the reason and contact person for data requests.

Please do so.

• Tables, Figures, and Formatting: Ensure that every table has clear titles; defined abbreviations; Correct totals and percentages; Appropriate footnotes explaining subsamples (e.g., biopsy, bacteriology); Include figure captions stating data sources, map copyrights, and consent for images. Standardize formatting throughout (fonts, heading levels, spacing).

Please address these carefully.

• Language and Style: Simplify long sentences, especially in the Introduction and Discussion. Ensure consistent tense use (Methods in past tense; Results in present; Discussion in past/present).

• References: Update literature to include recent (2020–2024) works on harmful algal blooms, marine toxin exposure, and dermal toxicity. Standardize all references according to PLOS style (authors, year, title, journal, DOI). Verify that all in-text citations correspond to the reference list.

Reference list looks ok.
