# Supplementary material for: Investigation of an outbreak of acute algal-associated dermatoses among artisanal fishermen in Senegal: A one health approach
Source: PLOS Glob Public Health. 2026 Apr 24;6(4):e0006328. doi: 10.1371/journal.pgph.0006328 (PMC13108758; doi:10.1371/journal.pgph.0006328)
Supplement: Attachment — Submitted filename: Response_to_Reviewers_auresp_3.docx [file pgph.0006328.s009.docx]

**Dr. Mbouna Ndiaye**

**MD, MPH, Field Epidemiologist**

Field Epidemiology Training Program (FETP) Coordinator,

Dakar, Senegal

Phone (+221) 651 94 85

Watsap: (+221) 70 960 74 23

[mbounandiaye@hotmail.fr](mailto:mbounandiaye@hotmail.fr)	Dakar, 21 Feb 2026

**Response Letter to Reviewers**

**Subject**: Response to reviewers

*Ref.: PGPH-D-25-01888*

*"* **Investigation of an Outbreak of Acute Algae-Associated Dermatoses in Artisanal Fishers in Senegal: A Unique Health Approach***"*

We provide you with point-by-point responses to the concerns raised in the form of a table (see below).

We have attached to this letter:

- An annotated copy of the manuscript that highlights the changes made to the original version. It was uploaded in a separate file entitled " **Revised Manuscript with Track Changes** ".
- An uncorrected version of my revised document with no track of changes. It was uploaded in a separate file entitled "**Manuscript**".

| **Issues raised** | **Actions taken**  **Nota Bene: the lines mentioned in this column refer to the new corrected version entitled "Manuscript"** |
| --- | --- |
| 1) **If the authors have sufficiently responded to your comments raised in a previous review phase and you feel that this manuscript is now acceptable for publication, you can indicate here that to bypass the "Comments to Author" section, enter your conflict of interest statement in the "Confidential to Editor" section, and submit your "Accept" recommendation.** | |
| **Criticism #1** : All comments have been taken into account |  |
| **Criticism #2** : (No answer) |  |
| 2) **Does this manuscript meet the** [**publication criteria**](https://emea01.safelinks.protection.outlook.com/?url=https%3A%2F%2Ftrack.editorialmanager.com%2FCL0%2Fhttps%3A%252F%252Fjournals.plos.org%252Fglobalpublichealth%252Fs%252Fcriteria-for-publication%2F1%2F010f019c071dbb03-7b0e5b34-1257-4fea-b0da-8ff68c07c9d4-000000%2FXOK75lduNMczGCPkW_eqqcOSD_32yNGcTSR4JVNF-3M%3D248&data=05%7C02%7C%7C4ae949fe6e9f4f842bc908de5ecbf1de%7C84df9e7fe9f640afb435aaaaaaaaaaaa%7C1%7C0%7C639052427507681193%7CUnknown%7CTWFpbGZsb3d8eyJFbXB0eU1hcGkiOnRydWUsIlYiOiIwLjAuMDAwMCIsIlAiOiJXaW4zMiIsIkFOIjoiTWFpbCIsIldUIjoyfQ%3D%3D%7C0%7C%7C%7C&sdata=mhX2Cnsa5pJ3J4hCS7bvje8r7Ih%2BTDLYBXQ5dWE89ss%3D&reserved=0) **of PLOS Global Public Health? Is the manuscript technically sound, and does the data support the conclusions? The manuscript should describe methodologically and ethically rigorous research with appropriate conclusions based on the data presented.** | |
| **Review #1** : Yes |  |
| **Criticism #2** : Yes |  |
| 3) **Was the statistical analysis carried out appropriately and rigorously?** | |
| **Review #1** : Yes |  |
| **Criticism #2** : I don't know |  |
| 4) **Have the authors made all the data underlying their manuscript results fully available (please see the data availability statement at the beginning of the manuscript PDF file)?** *The* [*PLOS data policy*](https://emea01.safelinks.protection.outlook.com/?url=https%3A%2F%2Ftrack.editorialmanager.com%2FCL0%2Fhttps%3A%252F%252Fjournals.plos.org%252Fglobalpublichealth%252Fs%252Fdata-availability%2F1%2F010f019c071dbb03-7b0e5b34-1257-4fea-b0da-8ff68c07c9d4-000000%2FShGJj3gbm5QbrV1xzhhQeQFejWAh9fWV58NEFB0p6nY%3D248&data=05%7C02%7C%7C4ae949fe6e9f4f842bc908de5ecbf1de%7C84df9e7fe9f640afb435aaaaaaaaaaaa%7C1%7C0%7C639052427507710205%7CUnknown%7CTWFpbGZsb3d8eyJFbXB0eU1hcGkiOnRydWUsIlYiOiIwLjAuMDAwMCIsIlAiOiJXaW4zMiIsIkFOIjoiTWFpbCIsIldUIjoyfQ%3D%3D%7C0%7C%7C%7C&sdata=61V7u0ODKWo%2B09Wj%2BvJCy6QhSMVe4JdTEvmnuSxtG5w%3D&reserved=0) *requires authors to make all data underlying the results described in their manuscript fully accessible without restriction, with rare exceptions. The data must be provided as part of the manuscript or its supporting information, or deposited in a public repository. For example, in addition to summary statistics, the data points behind the means, medians, and variance measures should be available. If there are restrictions on public data sharing — for example, participant privacy or third-party use of data — these should be specified.* | |
| **Criticism #1** : No | The declaration of data availability has been signed and attached in the submission system. In addition, the files *underlying the described results have been attached to the submission system; these are the files entitled: "Data Analysis", "Data Output Analysis Epi Info"* |
| **Criticism #2** : Yes |  |
| 5) **Is the manuscript presented intelligibly and written in standard English?** *PLOS Global Public Health does not edit accepted manuscripts, so the language of submitted articles must be clear, correct, and unambiguous. Any typos or grammar errors should be corrected during the review, so please note any specific errors here.* | |
| **Review #1** : Yes |  |
| **Criticism #2** : Yes |  |
| 6) **Please** *use the space available to explain your answers to the above questions. You can also include additional comments for the author, including concerns about dual publication, research ethics, or publication ethics. (Please upload your notice as an attachment if it exceeds 20,000 characters)* | |
| 1. 2. 3. 4. 5. 6. 6.1. Lines 27-30: The context of the abstract suggests that the causative agent (Vulcanodinium rugosum) was already known at the beginning of the study, which raises questions of coherence and chronology for me. In my view, the name of the causative agent should not appear in the background except as a hypothesis. On the contrary, it should appear as one of the results of the surveys. I also think that the identification of the causative agent should be added to the focus of the study. I suggest that the authors delete the first sentence of the summary or replace it with the one found in lines 65 to 67 of the introduction section. It would be useful to specify the nature of the alert for this mysterious disease as probably a dermatosis caused by fungi. I suggest this, and the authors should be free to improve: in November 2020, an alert about a "mysterious skin disease" was issued to fishermen. Fishermen are particularly vulnerable to skin diseases, particularly those caused by algae, due to their constant contact with seawater, fish, shellfish and fishing gear that may contain harmful agents. The study aimed to examine the alert, identify the causative agent, and propose preventive and control measures. | The causative agent (*V. rugosum*) was moved from the context to the results. Agent identification has been added to the objectives.  Proposal for amendment to the summary accepted and integrated (Line 27 – 31) |
| 6.2. Lines 31-34 In my opinion, the methodology remains quite poor. It should provide a little more detail on sampling (study population, inclusion criteria, selection), data collection (where, how, etc.), sampling for bacteriological analyses, etc. The section should indicate that both qualitative and quantitative approaches were used. | It's the limited number of words for the summary that prevents certain details. Nevertheless, we have added  that qualitative and quantitative approaches were used as you suggested (Line 33 - 34) |
| 6.3. Line 47: why "probable" when the algae have been isolated?  In the methodology section, participants for the qualitative approach (20), bacteriological test (10) and biopsy (4) were randomly selected. Authors should explain how they selected these sample sizes and discuss any limitations related to the use of small groups. | I had put the term "confirmed" but other reviewers proposed the term "probable" out of caution.  The use of this small group responds to a problem of resources. This limit was added in the chapter "study limit" (line 407-408) |
| 6.4. Lines 181-183: The authors indicate that the chi-square test was used to estimate the odds ratio (OR). However, only one transaction transaction is reported in the results section (lines 257-258). It is recommended that all results of bivariate analyses be presented in a table. Out of curiosity: why didn't they consider going further with multivariate analyses? | This is the only analysis that we have considered relevant. All other bivariate analyses were not performed |
| 6.5. In the methodology section, participants for the qualitative approach (20), bacteriological test (10) and biopsy (4) were randomly selected. Authors should explain how they determined these sample sizes and discuss the limitations associated with the use of small groups. | Added explanation of sample size determination and discussion of small group limitations ("study boundary" paragraph (line 407-408) |
| 6.6. Add a sentence summarizing the public health implications in the Conclusion.   This has not been addressed. I suggest adding something similar to this note:  "From a public health perspective, this study highlights how effective surveillance, coupled with evidence-informed action, and community outreach, can help reduce the impacts of outbreaks at the intersection of environmental and occupational health." | Addition of a sentence highlighting the importance of epidemiological surveillance and evidence-informed action. (Line 431-434) |
| 6.7. Population denominator and attack rate: Justify the use of the regional population as the denominator of the attack rate. Provide an alternative calculation using the exposed fishing population (e.g., 10,200 anglers) and report both rates. This comment was not addressed. Please add something like this: Attack rate with regional population as the denominator \| Attack rate with the exposed fishing population as the denominator; Please do the math and report. \| 5.4% Explicitly remove the numerator/denominator values in the Results section, and report only percentages. This comment was not addressed. Example: A total of 555 cases were diagnosed with an attack rate of 5.4% (555/10,200) among anglers and no deaths were reported. This should be corrected as follows A total of 555 cases were diagnosed with an attack rate of 5.4% among fishermen and no deaths were reported. | The general population was not used. The Attack Rate was calculated based on the exposed fishing population (10,200), i.e. an AT of 5.4%. (Line 204-205)  Correction made |
| 6.8. Provide details on laboratory protocols (sample preparation, instruments, analytical methods, detection limits, quality controls, interlaboratory validation with IFREMER).   This has not been addressed. I suggest adding an appendix with those details. | An appendix has been added including preparation, instrumentation and validation |
| 6.9. Toxicology and identification of Vulcanodinium rugosum: develop methods for the detection of toxins and identification of organisms: specify techniques (e.g., LC-MS/MS, microscopy, PCR), reference standards and validation procedures. Clarify the chain of custody and sample storage conditions.   See my comment above. These details can be added as an appendix. This will increase the rigor of your work and usefulness for those who wish to follow the same steps during a new outbreak. | Specification of detection techniques (LC-MS/MS, PCR) and validation procedures in appendix  The storage conditions and chain of custody have been clarified in the annex. |
| 6.10. Statistical and epidemiological reports: Ensure that all tables and texts include 95% confidence intervals and p-values for effect estimates (e.g., use of PPE vs. severity).   This has been partially addressed. Suggest adding the confidence interval to Table 1, for example. | The 95% confidence intervals have been added to Table 1, among others. |
| 6.11. Qualitative component: include a brief subsection Qualitative Methods: describe the selection of participants, the interview/focus group process, data transcription, coding, and the method of thematic analysis.   The following aspects were not addressed: data transcription, coding and the thematic analysis method.  Please mention at least briefly. | Added clarification on transcription, coding and the thematic analysis method. (Line 154-164) |
| 6.12. Present a short table summarizing key themes or illustrative quotes related to psychosocial and professional impact.   This comment was not addressed. | Insertion of a table summarizing the key themes of psychosocial and professional impact (in Appendix) |
| 6.13. Recognize and develop the limitations of the study (sampling bias, lack of quantification of exposure, recall bias, lack of control group).   Some of these points have been corrected.  Please respond to the following points as suggested by the reviewer:  Lack of exposure quantification, recall bias | The lack of quantification of exposure was added as a source of bias within the study boundaries. (Line 408 - 409)  However, the recall bias was not retained, given the short interval between the appearance of the first cases and the detection of the epidemic. The accuracy of fishermen's statements regarding the chronology of their symptoms is not significantly affected |
| 6.14. Ethical Considerations and Image Clearances: Provide full details of the ethics approval (name, number, and date of the IRB). Explain how informed consent was obtained (and parental consent if minors are included). Confirm that patient images have been masked/anonymized and used with consent or removed appropriately.   This has been partially addressed.  I assume that approval by the ethics committee was not feasible due to the urgent nature of the study. In this case, please indicate that approval by the Rehabilitation Committee was not feasible. If obtained, please indicate from where and the number. The fact that the study is commissioned by the Ministry of Health does not remove the need for IRB approval. However, if it were part of routine service delivery, you could probably justify it to some extent. Please have a sentence specifically about the photographs. "Patient images were masked when identifying details were visible. Consent was obtained to submit photographs in a non-identifiable format for educational purposes. If this has not been done, explain why it has not been done. | All the details of the ethics approval (name, number and date of the IRB) are already provided and attached in the electronic system.  I don't understand when you ask me to explain "how informed consent was obtained (and parental consent if minors are included)". What additional details would you like to put in the manuscript?  Confirmation that patient images have been masked/anonymized and used with consent. |
| 6.15. Data Availability: - Provide a complete statement of data availability required by PLOS: indicate where the dataset and supporting files can be accessed (repository name, DOI/URL). If access is restricted, specify the reason and contact person for data requests.   Please do it. | A complete PLOS compliant declaration has already been provided and attached in the electronic submission system |
| 6.16. Tables, Figures, and Formatting: Make sure each table has clear titles; defined abbreviations; Correct totals and percentages; Appropriate footnotes explaining subsamples (e.g., biopsy, bacteriology); Include figure captions indicating data sources, map copyright, and image consent. Standardize the layout across all areas (fonts, header levels, spacing).   Please treat these questions carefully. | Standardization of the PLOS style, |
| 6.17. Language and style: Simplify long sentences, especially in the introduction and discussion. Make sure you use tenses consistently (Past tense methods; Results at the moment; Past/present discussion). | Simplification of sentences and appropriate use of tense |
| 6.18. References: Update the literature to include recent work (2020–2024) on harmful algal blooms, exposure to marine toxins, and dermal toxicity. Standardize all references according to the PLOS style (authors, year, title, journal, DOI). Verify that all citations in the text match the list of references.   The list of references seems correct. | Verification done |
