# Supplementary material for: Investigation of an outbreak of acute algal-associated dermatoses among artisanal fishermen in Senegal: A one health approach
Source: PLOS Glob Public Health. 2026 Apr 24;6(4):e0006328. doi: 10.1371/journal.pgph.0006328 (PMC13108758; doi:10.1371/journal.pgph.0006328)
Supplement: Attachment — Submitted filename: Response_to_Reviewers_auresp_4.docx [file pgph.0006328.s010.docx]

**Dr. Mbouna Ndiaye**

**MD, MPH, Field Epidemiologist**

Field Epidemiology Training Program (FETP) Coordinator

Dakar, Senegal

Phone (+221) 651 94 85

Watsap: (+221) 70 960 74 23

[mbounandiaye@hotmail.fr](mailto:mbounandiaye@hotmail.fr)	Dakar, 13 March 2026

**Response Letter to Reviewers**

**Subject**: Response to reviewers

*Ref.: PGPH-D-25-01888*

*"* **Investigation of an Outbreak of Acute Algae-Associated Dermatoses in Artisanal Fishers in Senegal: A Unique Health Approach***"*

We provide you with point-by-point responses to the concerns raised in the form of a table (see below).

We have attached to this letter:

- An annotated copy of the manuscript that highlights the changes made to the original version. It was uploaded in a separate file entitled " **Revised Manuscript with Track Changes** ".
- An uncorrected version of my revised document with no track of changes. It was uploaded in a separate file entitled "**Manuscript**".

| **Issues raised** | **Actions taken**  **Nota Bene: the lines mentioned in this column refer to the new corrected version entitled "Manuscript"** |
| --- | --- |
| 1. Population denominator and attack rate: Justify the use of the regional population as the denominator of the attack rate. Provide an alternative calculation using the exposed fishing population (e.g., 10,200 anglers) and report both rates. This comment was not addressed. Please add something like this: Attack rate with regional population as the denominator \| Attack rate with the exposed fishing population as the denominator; Please do the math and report. \| 5.4% Explicitly remove the numerator/denominator values in the Results section, and report only percentages. This comment was not addressed. Example: A total of 555 cases were diagnosed with an attack rate of 5.4% (555/10,200) among anglers and no deaths were reported. This should be corrected as follows A total of 555 cases were diagnosed with an attack rate of 5.4% among fishermen and no deaths were reported. | Both rates were reported in text form (Line 205-207).  The correction was made in text form because the number of tables is limited by the journal. (Line 205-207).  All numerator/denominator values have been suppressed  Correction made (Line 205). |
| 2. Provide details on laboratory protocols (sample preparation, instruments, analytical methods, detection limits, quality controls, interlaboratory validation with IFREMER).   This has not been addressed. I suggest adding an appendix with those details. | Dear reviewer, maybe you don't have the right version of the manuscript. Since your first remark, the file has been added as an appendix 1 |
| 3. Toxicology and identification of Vulcanodinium rugosum: develop methods for the detection of toxins and identification of organisms: specify techniques (e.g., LC-MS/MS, microscopy, PCR), reference standards and validation procedures. Clarify the chain of custody and sample storage conditions.   See my comment above. These details can be added as an appendix. This will increase the rigor of your work and usefulness for those who wish to follow the same steps during a new outbreak. | Dear reviewer, maybe you don't have the right version of the manuscript. Since your first remark, the file has been added as an appendix 2  Specification of detection techniques (LC-MS/MS, PCR) and validation procedures in Appendix 2  Storage conditions and chain of custody have been clarified in Appendix 2 |
| 4. Statistical and epidemiological reports: Ensure that all tables and texts include 95% confidence intervals and p-values for effect estimates (e.g., use of PPE vs. severity).   This has been partially addressed. Suggest adding the confidence interval to Table 1, for example. | Dear reviewer, maybe you don't have the right version of the manuscript. Since your first remark, the 95% confidence intervals have been added, among other things, to Table 1. (Line 237). |
| 5. Qualitative component: include a brief subsection Qualitative Methods: describe the selection of participants, the interview/focus group process, data transcription, coding, and the method of thematic analysis.   The following aspects were not addressed: data transcription, coding and the thematic analysis method.  Please mention at least briefly. | Added clarification on transcription, coding and the thematic analysis method. (Line 154-164)) |
| 6. Present a short table summarizing key themes or illustrative quotes related to psychosocial and professional impact.   This comment was not addressed. | Dear reviewer, maybe you don't have the right version of the manuscript. Since your first remark, the file has been added as an appendix 3 |
| 7. Recognize and develop the limitations of the study (sampling bias, lack of quantification of exposure, recall bias, lack of control group).   Some of these points have been corrected.  Please respond to the following points as suggested by the reviewer:  Lack of exposure quantification, recall bias | The absence of a comparison group (e.g. case-control study) and the absence of quantification of exposure were added as a source of bias within the study limits. (Line 407 - 411)  However, the recall bias was not retained, given the short interval between the appearance of the first cases and the detection of the epidemic. The accuracy of fishermen's statements regarding the chronology of their symptoms is not significantly affected |
| 8. Ethical Considerations and Image Clearances: Provide full details of the ethics approval (name, number, and date of the IRB). Explain how informed consent was obtained (and parental consent if minors are included). Confirm that patient images have been masked/anonymized and used with consent or removed appropriately.   This has been partially addressed.  I assume that approval by the ethics committee was not feasible due to the urgent nature of the study. In this case, please indicate that approval by the Rehabilitation Committee was not feasible. If obtained, please indicate from where and the number. The fact that the study is commissioned by the Ministry of Health does not remove the need for IRB approval. However, if it were part of routine service delivery, you could probably justify it to some extent. Please have a sentence specifically about the photographs. "Patient images were masked when identifying details were visible. Consent was obtained to submit photographs in a non-identifiable format for educational purposes. If this has not been done, explain why it has not been done. | All details of the ethics approval (name, number and date of the IRB) are already provided and attached in the electronic system as a separate file to the manuscript. This has been required by the newspaper since the beginning of the submission  (reference n°000698/MSAS/DGS/DP of 9 October 2020). Nevertheless, the clarification has been made (line 193)  Confirmation that patient images have been masked/anonymized and used with consent (line 196) |
| 9. Data Availability: - Provide a complete statement of data availability required by PLOS: indicate where the dataset and supporting files can be accessed (repository name, DOI/URL). If access is restricted, specify the reason and contact person for data requests.   Please do it. | A complete PLOS-compliant declaration has already been provided and attached in the electronic submission system. The clarification was made on line 442-445 |
| 10. Tables, Figures, and Formatting: Make sure each table has clear titles; defined abbreviations; Correct totals and percentages; Appropriate footnotes explaining subsamples (e.g., biopsy, bacteriology); Include figure captions indicating data sources, map copyright, and image consent. Standardize the layout across all areas (fonts, header levels, spacing).   Please treat these questions carefully. | Standardization of the PLOS style, |
| 11. Language and style: Simplify long sentences, especially in the introduction and discussion. Make sure you use tenses consistently (Past tense methods; Results at the moment; Past/present discussion). | Simplification of sentences and appropriate use of tense |
| 12. References: Update the literature to include recent work (2020–2024) on harmful algal blooms, exposure to marine toxins, and dermal toxicity. Standardize all references according to the PLOS style (authors, year, title, journal, DOI). Verify that all citations in the text match the list of references.   The list of references seems correct. | Verification done |
